# Supplementary material for: The non-ELR CXC chemokine encoded by human cytomegalovirus UL146 genotype 5 contains a C-terminal β-hairpin and induces neutrophil migration as a selective CXCR2 agonist
Source: PLoS Pathog. 2022 Mar 10;18(3):e1010355. doi: 10.1371/journal.ppat.1010355 (PMC8939814; doi:10.1371/journal.ppat.1010355)
Supplement: S1 Fig — The secondary structures of CXCL8 and vCXCL1GT1-14 are shown in light blue (β-strands) and red (α-helices) as determined by NMR and X-ray structures for CXCL8 [22–24] and by Rosetta modelling for vCXCL1GT1-GT14. (PDF) [file ppat.1010355.s001.pdf]

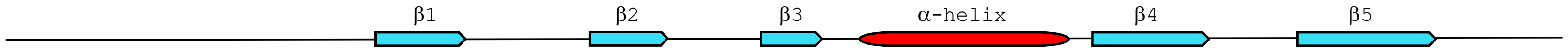


```
CXCL8   AVLPRSAKELRCQCIKTYSK-PF--HPKFIKELRVIESG-PHCANTEIIVKLS----DGRELCLDPKENWVQRVVEKFLKRAENS-------------------------------------

GT1     -------TELRCRCLHR-KW-PPNKIILGNYWLHRDPRG-PGCDKNEHLLYPDGRKPPGPGVCLSPDHLFSKWLDKHNDNR----WYNVNITKSP-------GPRRINITLIGVRG------
GT2     -------TELRCKCLDGKKL-PPKTIMLGNFWFHRESGG-PRCNNNEYFLYLGGGKKHGPGVCLSPHHPFSKWLDKRNDNR----WYNVNVTRQPE-----RGPGKITVTLVGLKE------
GT3     -------TELRCKCPGDKKL-PRTRIMLGDFWTHRESGG-PGCNGYQYLLYFNNGGKHGRGVCLAPDHHISKWLDTHNDGR----WYNVNITKQPGRRTGGRGPGQVNITLIAVKQ------
GT4     -------TELRCKCAGGQSWHPRGKWPTKHHWLECYPPS-GNCPAGELLIYFEEH--NWSPKCVHVHNPFGQKFMSKCDKHE---WFEVTFNSTR------KYPMITRKGSTKPTFSSGK--
GT5     --GFGTEGNGRCTCIGYHRLL--GQLPRGTFWLGHLPPG-SHCPKGQVMIKIG----QGPIVCLSDYHPLSKWMYGNHKSGSET-WLQIKMEG----------PRNATVVQRSNTRP-----
GT6     --GLGSEGNGRCTCVGYHRFD--KQLPRGTIWLGHRPPG-PHCPRGDVLMKLG----EQPTVCLSDHHPLSKWMYRHHGSDTEI-WFQIEFKG----------PQNTKVVSKSFTPPS----
GT7     -------RELRCPCTHKALHHP----IGGLFWVGRDPPNPPECDKPQHYLLPP----RGKPVCLAPDHHLSKWLDGKKDNS----WHRVLVKVKDS-----NGPHVEENAVTNKRPRWK---
GT8     -------VELRCRCS-NGSNHP----VFGVFWVGYKPPD-PTCDKTQHFLLPP----RQTPVCLSPDHYLSKWVDGKRSNW----WHKVFIKKNSD-----NGPHIEDKSDTNRHPPWRL--
GT9     -------VELRCPCGSNGLSYP----IGGFFWIGYNPPDPPKCEKPQHFLLPP----KGKPVCLSPDHVLSKWLHGKSSNT----WHKVLLRTKGG-----GGPHVEERTASNGRPPWKLKF
GT10    -------VELRCPCGSNSVNKP----VSGVFLIGRDPPNPPGCNRFQYYLAPP----HGKPVCLDSEHHISKWLDGQNSNS----WYKVIIKNGDD-----NKPKVEKRTEIKKRFKWN---
GT11    -------VELRCPCGSNGLRNP----LSGVFLVGRDPPRPPGCTKHQHYLVPP----NGRRACLNPDHHLSKWLDAQNSNS----WYKVVVTTGDG-----GGPHVDKKAEIKKRLS-----
GT12    -------VELRCPCT-DGFRNP----LFGLFLIGHDPPHPPVCERDQFFLKPP----QGKTVCLGPEHHLSSWLKGQNSSL----WHKVLVTKKGD------EPQVQKRGEYPKNRARIIV-
GT13    -------TELRCPCT-NGLHDP----LYGIFYAGRDPPRPPGCEKDQYYLKPP----KGKAVCLGPHHHLSIWLNGQNSSL----WHKVLVTGKNGN-----GPHVTKKGDFPRGRKNIMI-
GT14    -------VELRCPCTHNVLNRP----IGGVFWIGRDPPKPPECDKPQHFLLTS----QGKTVCLGPDHHITKWLDGQNSNS----WYKVFITTNGN-----NGPQIHKRAEHNKRPKWKL—
```

S1 Fig. Supplemental alignment showing the exact location of CXCL8 and vCXCL1 secondary structures. The secondary structures of CXCL8 and vCXCL1$_{GT1\text{-}14}$ are shown in light blue (β-strands) and red (α-helices) as determined by NMR and X-ray structures for CXCL8 [23-25] and by Rosetta modelling for vCXCL1$_{GT1\text{-}GT14}$.
