# Supplementary material for: The non-ELR CXC chemokine encoded by human cytomegalovirus UL146 genotype 5 contains a C-terminal β-hairpin and induces neutrophil migration as a selective CXCR2 agonist
Source: PLoS Pathog. 2022 Mar 10;18(3):e1010355. doi: 10.1371/journal.ppat.1010355 (PMC8939814; doi:10.1371/journal.ppat.1010355)
Supplement: S2 Fig — Samples of vCXCL1GT1 (A) and vCXCL1GT5 (B) were digested with trypsin followed by mass spectroscopy. (PDF) [file ppat.1010355.s002.pdf]

S2 Fig. Peptide mass fingerprint of vCXCL1$_{GT1}$ and vCXCL1$_{GT5}$

Samples of vCXCL1$_{GT1}$ (A) and vCXCL1$_{GT5}$ (B) digested with trypsin followed by mass spectroscopy.

A

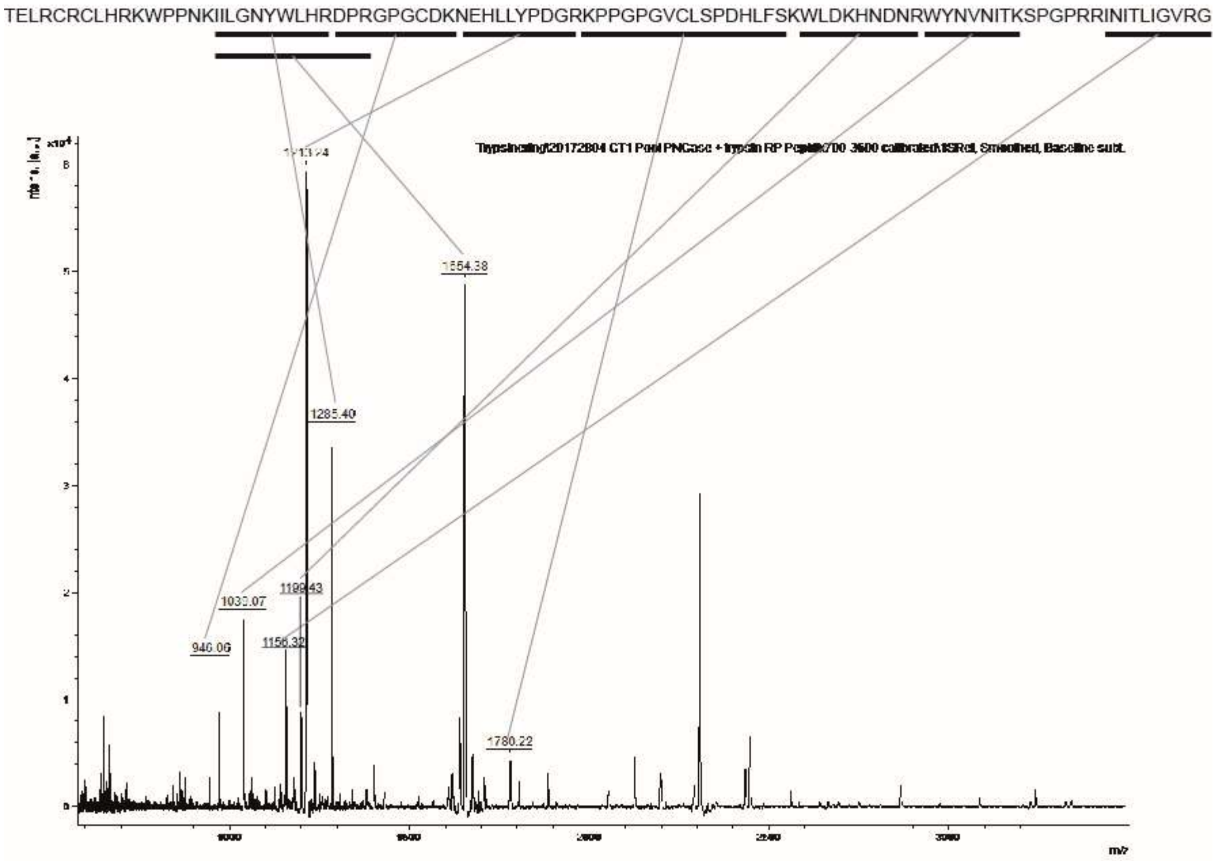

vCXCL1$_{GT1}$ trypsin fragments			
Mass	Sequence	Expected mass (neutral)	Difference (Da)
946,06	DPRGPGCDK	943,42	1,64
1039,07	WYNVNITK	1036,53	1,54
1156,32	INITLIGVRG	1153,73	1,59
1199,43	WLDKHNDNR	1196,57	1,86
1213,24	NEHLLYPDGR	1212,59	-0,35
1285,40	IILGNYWLHR	1283,71	0,69
1654,37	IILGNYWLHRDPR	1651,89	1,48
1780,22	KPPGPGVCLSPDHLFSK	1777,92	1,30
**Coverage**	**76,84 %**		

B

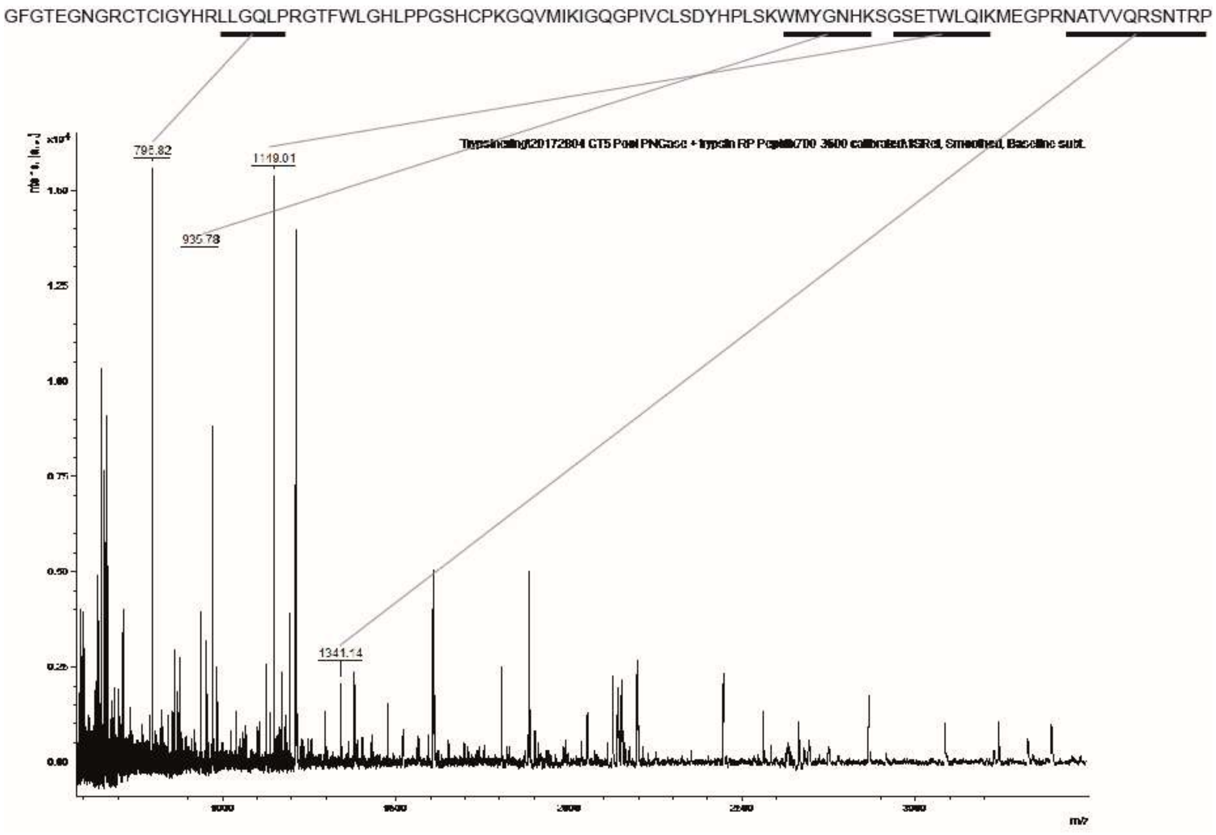

vCXCL1$_{GT5}$ trypsin fragments			
Mass	Sequence	Expected mass (neutral)	Difference (Da)
796,82	LLGQLPR	795,50	0,32
935,78	WMYGNHK	934,41	0,37
1149,01	SGSETWLQIK	1147,59	0,42
1341,14	NATVVQRSNTRP	1341,71	-1,57
**Coverage**	**37,11 %**		
