# Supplementary material for: The non-ELR CXC chemokine encoded by human cytomegalovirus UL146 genotype 5 contains a C-terminal β-hairpin and induces neutrophil migration as a selective CXCR2 agonist
Source: PLoS Pathog. 2022 Mar 10;18(3):e1010355. doi: 10.1371/journal.ppat.1010355 (PMC8939814; doi:10.1371/journal.ppat.1010355)
Supplement: S3 Fig — (A) The top model (lowest Rosetta total score) is shown in an overlay for each genotype with the extended C-termini hidden for clarity. The average Cα-RMSD of core residues is 3.6 Å. All models adopt the canonical chemokine core structure. (B) The top models for each genotype are displayed individually. Each C-terminus forms a β-hairpin except for vCXCL1GT3, vCXCL1GT13, and vCXCL1GT14. (PDF) [file ppat.1010355.s003.pdf]

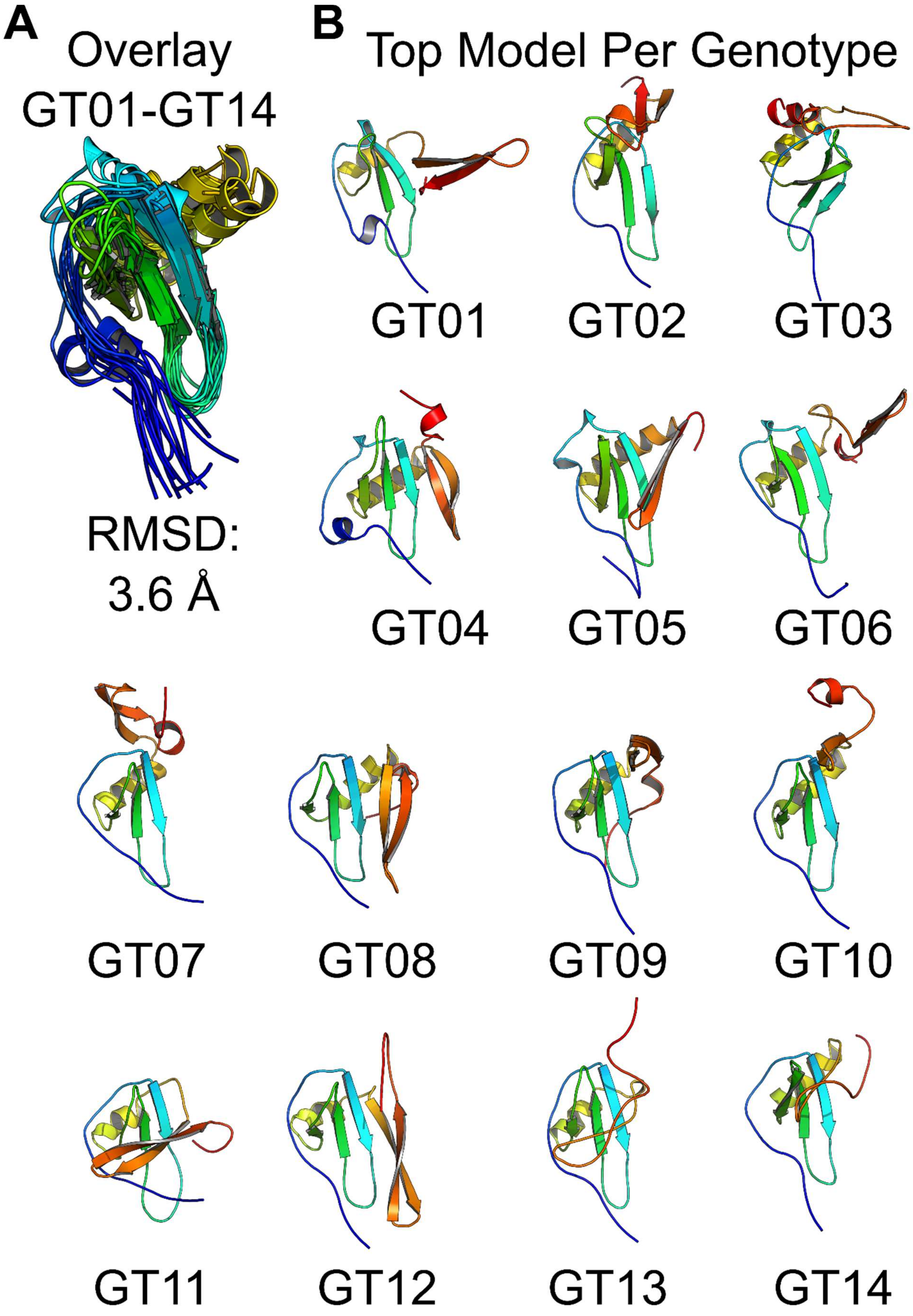
A
Overlay
GT01-GT14
RMSD:
3.6 Å
B
Top Model Per Genotype
GT01
GT02
GT03
GT04
GT05
GT06
GT07
GT08
GT09
GT10
GT11
GT12
GT13
GT14

**S3 Fig. Top models for each UL146 genotype.**

**(A)** The lowest scoring model by Rosetta total energy is shown in an overlay for each genotype with the extended C-termini hidden for clarity. The average Cα-RMSD of core residues is 3.6 Å. All models adopt the canonical chemokine core structure. **(B)** The lowest scoring models for each genotype are displayed individually. Each C-terminus forms a β-hairpin except for $vCXCL1_{GT3}$, $vCXCL1_{GT13}$, and $vCXCL1_{GT14}$.
