# Supplementary material for: The non-ELR CXC chemokine encoded by human cytomegalovirus UL146 genotype 5 contains a C-terminal β-hairpin and induces neutrophil migration as a selective CXCR2 agonist
Source: PLoS Pathog. 2022 Mar 10;18(3):e1010355. doi: 10.1371/journal.ppat.1010355 (PMC8939814; doi:10.1371/journal.ppat.1010355)
Supplement: Attachment — Submitted filename: 211226 vCXCL1 Response to reviewers.docx [file ppat.1010355.s005.docx]

**Answers to the reviewers:**

***Reviewer 1:***

***Part I - Summary:*** *The manuscript by Luttichau is a well written and comprehensive analysis of the various chemokines expressed by human cytomegalovirus (HCMV). The UL146 gene exhibits a high degree of genetic diversity based on sequencing of various clinical and laboratory virus strains. The authors provide a detailed evaluation of the sequence and structure, focusing on two variants, designated vCXCL1GT1 and vCXCL1GT2. In addition, functional analysis was performed using a variety of signaling assays, which is a strength of the study. Signaling assays included IP accumulation, beta arrestin recruitment, and cell migration. Overall, the work is soundly presented and well controlled, and the results provide an important contribution to the literature by illustrating how subtle differences in sequence and* *structure can be accompanied by significant changes in function and downstream signaling.*

***Authors reply 1.1:***

We are happy to hear that reviewer 1 finds our manuscript well written, the analysis of the two genotypes of the CMV chemokine comprehensive, and that the manuscript provides an important contribution to the literature.

**Part II - Key experiments required for acceptance**: No response

**Part III – Minor Issues: Editorial and Data Presentation Modifications:**

1. *Line 60 refers to UL146 as a cytokine, whereas the title and majority of the manuscript use the term cytokine. While a chemokine is a type of cytokine, this term seems unnecessarily confusing and inconsistent here.*

***Authors reply 1.2:***

In response to the reviewer’s comment, we have replaced “cytokine” with “chemokine”.

*2. Introduction lines 74-76 – references should be provided*

***Authors reply 1.3:***

A reference has been added as requested.

*3.* *Figure 1 - clarify what black and gray shading represent*

***Authors reply 1.4:***

White on black background represents identical residues, and black on grey background represents similar residues, which share characteristics such as being acidic, basic, aromatic or hydrophobic. Therefore, the following line has been added in the legend to figure 1:

*“White on black illustrates identical residues, and black on grey similar residues.”*

*4. Fig 2 – suggest including small schematic to depict differences between two chemokine (glycosylation sites and additional 9 peptides)*

***Authors reply 1.5:***

We thank reviewer 1 for the suggestion to improve figure 2. We have inserted a *new panel* 2A depicting the 14 genotypes and the approximate locations of the predicted N- and O-glycosylation sites.

*5.* *Line 226 – “roughly half” – give exact numbers*

***Authors reply 1.6:***

Wild-type vCXCL1_GT5_ was 60% efficacious and the truncated variants ~30%, which is specified in the manuscript. To accommodate the request, we have deleted “roughly” and specified the individual variant efficacies.

6. *Line 438 – clarify what PDB: 6LFO means*

***Authors reply 1.7:***

We would like to point the attention of reviewer 1 to line 30 *under abbreviations*, where PDB has been explained as the short and commonly used term for Protein Data Bank. 6LFO is the structure of CXCL8 bound to CXCR2 deposited in the protein databank. In order to clarify we have changed the wording to PDB ID: 6LFO.

*7. Line 731 – clarify cell number*

***Authors reply 1.8:***

Reviewer 1 wants a clarification of “cell number” in line 731. Line 731-32 reads

“*incubated for 1.5 h at 37°C and 5% CO_2_. For stably transfected L1.2 cells, 75 µl cell suspension containing 150.000 cells in RPMI1640 + 0.5% BSA medium were added per well and incubated”*.

We are unsure what the reviewer means by “cell number” as the cell count is already specified in the referenced sentence.

***Reviewer 2:***

***Part I - Summary:*** *This manuscript describes the unique folding in the COOH terminus of the viral chemokines from HCMV (i.e., in the title), however the majority of the paper is about the longer/shorter versions of the amino terminus. There are interesting findings such that the non ELR motif (i.e., NGR instead of ELR) in genotype GT5 (although GT6 as well) could stimulate CXCR2 and b arrestin signaling. Also the finding that the longer amino terminus led to more activation (usually additions to the NH2 leads to less activation/antagonism such as the case with CCL5). The additional finding of this paper is that it “confirmed” the potential glycosylations via MALDI TOF.*

***Authors reply 2.1:***

We are happy to hear that reviewer 2 finds the activation of CXCR2 by a non-ELR viral chemokine and the unique folding of the C-terminal interesting.

*The major flaws of this manuscripts are:*

1) *In order to address the role of the COOH on function there should have been experiments that truncated/swapped the different COOH termini. Instead it includes only modeling of the COOH. It’s possible that this longer COOH could function only in vivo as part of the GAG binding to establish the chemotactic gradient, which may not be detectable in vitro.*

Authors:

Below we address the above comments in three separate responses to: (i) “only” modelling without in vitro experiments (**2.2)**; (ii) investigation of the function of the C-terminus by truncation (**2.3**); and (iii) function of the tail in GAG binding (**2.4**).

***Authors reply 2.2****:*

*In vitro data to support in silico models:*

As mentioned above, the main focus of this paper was to determine the receptor target of the non-ELR chemokine vCXCL1_GT5_ and to model the extraordinary long C-termini for these HCMV chemokines. While it is true that the C-terminal β-hairpin fold was only supported by *in silico* studies in the original manuscript, we find it striking that the ten top models of each of 11 genotypes predicted the same structure.

Still, we decided to perform NMR studies of vCXCL1_GT1_ to assign backbone chemical shifts of the extended C-terminus to see whether *in vitro* experiments would support the results of the Rosetta modelling. The results from TALOS+ analysis of the ^1^H, ^13^Cα, ^13^Cβ, ^13^C′, and ^15^N chemical shifts of the vCXCL1_GT1_ tail predicted β-strands for residues 72-79 and 85-94 with high confidence, matching the consensus location of the β-hairpin in the Rosetta models.

*Changes in the manuscript due to this reply follow after all our replies to the comments from the reviewers.*

***Authors reply 2.3****:*

*Function by truncation:*

We agree with reviewer 2 that experiments with C-terminal truncated vCXCL1s have potential to reveal the function of the C-terminus, and decided to include a tailless vCXCL1_GT1_ chemokine in our studies. Interestingly, the tailless vCXCL1_GT1_ chemokine was non-functional in contrast to the wild-type vCXCL1_GT1._ Furthermore, thermal denaturation using nano differential scanning fluorimetry of the wild-type and tailless vCXCL1_GT1_ revealed that the wild-type chemokine had a melting curve with an apparent Tm of 66.4 ˚C, while the tailless version was unfolded from 20-95 ˚C. Thus, one of the functions of the C-terminus of vCXCL1_GT1_ seems to be to stabilize the chemokine fold of the protein.

*Changes in the manuscript due to this reply follow after all our replies to the comments from the reviewers.*

***Authors reply 2.4****:*

*C-termini and GAG binding:*

We agree with reviewer 2 that determination of the function of the C-terminus is important and possibly relates to GAG-binding (but probably only for some of the genotypes as there is huge variation in number of basic residues in the tails of the different vCXCL1s, as seen in the new Table S1) or interaction with other proteins as we have mentioned in the discussion. We have added a section in the manuscript, where we discuss the function of and compare the extended C-termini of human and viral chemokines. GAG binding isn’t the focus of the present manuscript, but we are confident that GAG binding to the tails of vCXCL1s can be investigated by *in vitro* experiments in future projects as other groups have done so for human chemokines with extended C-termini (please see Table S1, and refs 22, 40 and 43).

*Changes in the manuscript due to this reply follow after all our replies to the comments from the reviewers.*

2) *The novelty of this manuscript is lessened in that it is similar to the studies completed by Heo et al which used full length UL146 ORF expressed in baculovirus from 11 different genotypes (including what was called TX15 with an NGR motif). This manuscript really only addressed a single viral chemokine (GT5) while GT6 has a similar non ELR motif. This is another flaw in this manuscript. In the Heo manuscript they did not find that NRG vCXCL1 proteins activation of b arrestin or G proteins but did see some slight activation via calcium flux and upregulation of adhesion proteins. This contradicts the findings of this paper but, as the authors pointed out, it could be due to differences in the NH2 from baculovirus vs the ones tested in this paper. It would have been nice to see a comparison between TX15 from that paper and GT5 and GT6.*

***Authors reply 2.5****:*

We are happy to be given the opportunity to discuss the differences/similarities between our manuscript and the report by Heo *et al.* (from the **groups of Tim E. Sparer and** **Martine Smit**) as both investigate the functional properties of this group of HCMV chemokines. However, we find that *important* and *crucial* differences in the research questions posed and research strategies chosen by each group also need to be addressed:

*Fundamental difference number 1:*

*Heo et al.* tested their eleven ligands in one functional assay on only CXCR2 and in binding on two receptors (CXCR1 and CXCR2). In contrast, we tested vCXCL1_GT5_ individually on all human chemokine receptors (CCR1, CCR2, CCR3, CCR4, CCR5, CCR6, CCR7, CCR8, CCR9, CCR10, CXCR1, CXCR2, CXCR3, CXCR4, CXCR5, CXCR6, XCR1, CX3CR1) and the CMV-encoded chemokine receptor US28. Thereby, we could show that vCXCL1_GT5_ did not target other chemokine receptors and selectively targeted CXCR2. Thus, we *do not* consider the focus on vCXCL1_GT5_ a flaw, but rather a test of our initial hypothesis.

*Fundamental difference number 2:*

In contrast to our finding that vCXCL1_GT5_ (and in the revised manuscript also the other non-ELR CMV chemokine vCXCL1_GT6_) are highly efficacious in functional assays on CXCR2, Heo *et al.* did not find any activity of vCXCL1_GT6_ (called Tx15) on CXCR2. While we don’t know the reason for the lack of function of vCXCL1_GT6_ on CXCR2 in the Heo paper, a hint could be in their figure 1, where they align the mature forms of the 11 vCXCL1s and show vCXCL1_GT6_ (Tx15) with a four residue N-terminus, which we find represents a truncated form.

In conclusion, we have for the first time *established* that non-ELR chemokines can indeed activate CXCR2.

*Fundamental difference number 3, 4 and 5:*

Additionally, we provide answers to important questions not raised by Heo *et al.* such as

i) the significance of N-terminal truncation,
ii) predicted glycosylation versus *in-vitro* glycosylation, and
iii) structure and function of the C-terminus by *in silico* and *in vitro* experiments.

*Fundamental difference number 6*:

Finally, the majority of the employed assays are unique for each research group, i.e. the number of overlapping assays are limited. In our view, the most important difference in use of assays is that Heo *et al.* to a great extent used primary cells such as PBNs and the primary neutrophil-like cell-line HL60 T2, while we primarily used cell-lines transfected with individual chemokine receptors. Each strategy can have its merits and drawbacks, and in our view, the two works supplement each other more than they overlap.

As a response to the reviewer’s comments we decided to expand the manuscript by expressing the other non-ELR chemokine vCXCL1_GT6_ and another ELR chemokine vCXCL1_GT4_ and test them in parallel with vCXCL1_GT1_ and vCXCL1_GT5_ in IP-accumulation assays on cells either expressing CXCR1 or CXCR2. vCXCL1_GT4_, vCXCL1_GT5_ and vCXCL1_GT6_ were found to activate CXCR2, but not CXCR1, while vCXCL1_GT1_ activated both receptors.

*Changes in the manuscript due to this reply follow after all our replies to the comments from the reviewers*.

3) *Another flaw is that this group used the bacterially expressed GT5 proteins in its functional assays (i.e., with a predetermined NH2 terminus and no glycosylations). Although their findings that the different NH2 termini length (i.e., longer functions better) is important, it would have been interesting to see whether this holds true for the other genotypes such as GT6 or the other GT with ambiguous signal cleavage sequences*.

***Authors reply 2.6****:*

First of all we do not consider the use of prokaryotically expressed chemokines a *“flaw”* although we agree with reviewer 2 that it is desirable to use a form of the protein that is as close as possible to its “in vivo” form.

- To generate sufficient amounts of eukaryotically expressed chemokines for cellular assays is difficult/impossible with few exceptions. Prokaryotically expressed recombinant proteins have been used in almost all articles reporting on chemokine function in Pubmed. Such studies have provided - and still provide - valuable results to illuminate the role and function of chemokines, and importantly have been published in very high impact journals. To provide an example, eight of the nine research groups that de-orphanized the members of the CXCR receptor family did *not* use eukaryotically expressed chemokines. Thus, only one of the nine groups used an eukaryotically expressed protein, which came with the expense of introducing a serious bias as the protein was expressed with a highly charged nine residue N-terminal FLAG-tag – please see table below for references.

- Moreover, we carefully established the signal peptide cleavage site of vCXCL1_GT5_ by expression in eukaryotic cells (followed by purification, deglycosylation and mass spec) before *E. coli* expression of recombinant vCXCL1_GT5_ in amounts that allowed for extensive testing. This procedure is rarely done.
- As the reviewers suggested testing of additional genotypes, we have made recombinant expression of vCXCL1_GT4_ and vCXCL1_GT6_. vCXCL1_GT4_ was expressed with a four residue N-terminus, which is predicted for 12 of 14 genotypes including vCXCL1_GT1_ (please see fig 1), and which we demonstrated is the correct N-terminus for vCXCL1_GT1_ by establishing the signal peptide cleavage site. vCXCL1_GT6_ was expressed with the predicted N-terminus GLGSEGNGR, which is very similar to the N-terminus of vCXCL1_GT5_ (GFGTEGNGR) that was determined experimentally in the present paper.
- The observation that shortening the N-terminus of vCXCL1_GT5_ results in decreased activity is in line with findings from studies on N-terminal truncation of other chemokines, where the agonistic activity has been shown to decrease – in some cases to the point where the truncated chemokine functions as an antagonist, as shown for CCL2, CCL5, CCL11, CXCL8, CXCL12 (reviewed by Fernandez and Lolis in Annual Review of Pharmacology and Toxicology, 2002). Therefore, we would not be surprised if truncation of the N-terminus of other vCXCL1 genotypes would result in decreased functional activity compared to the activity of the wild-type protein.

*Minor points:*

1) *In Fig. 7 is that the GT5 (1-97)? That needs to be clarified.*

***Authors reply 2.7:***

We have clarified that it is indeed the wild-type (1-97) vCXCL1_GT5_ in the legend for figure 7 and, for consistency, also for figure 6.

2) *In Fig. 7, it’s interesting that you show different bell shaped curves for the CXCR2 transfectants but similar ones with the neutrophils. That should be explored/explained.*

***Authors reply 2.8:***

The reason for the difference is that chemotactic responses are compared between different cell populations (L1.2 cell-line and primary neutrophils). The chemotactic response involves several steps of the cellular machinery following receptor activation. If one of these post-receptor-activation-steps is limiting the chemotactic response in neutrophils one could see overlapping chemotactic responses for neutrophils. Thus, we have added the following sentence in the description of the chemotaxis results:

“*The higher efficacy of vCXCL1_GT1_ in relation to vCXCL1_GT5_ to induce migration of CXCR2 transfected cells in contrast to the same efficacy for the two viral chemokines to induce migration of neutrophils is likely due to cell dependent differences in the chemotactic machinery resulting in saturation of specific steps in the signaling cascade leading to migration.*”

3) *Fig. 8, 9 are modeling but would be necessary to prove it with actual experiments to confirm the modeling.*

***Authors reply 2.9:***

We fully agree. In future projects it would be interesting to confirm the “*in silico*” results by NMR/crystal structures of vCXCL1_GT4_ (figure 9 in the revised manuscript) and vCXCL1_GT5_ bound to CXCR2 (figure 10 in the revised manuscript). In addition, a structure of vCXCL1_GT1_ or CXCL8 bound to CXCR1 would be enlightening.

**Part II - Key experiments required for acceptance:** No response

**Part III – Minor Issues: Editorial and Data Presentation Modifications:** No response

***Reviewer 3:***

***Part I - Summary:*** *Human cytomegalovirus (HCMV) encodes multiple chemokine and chemokine receptor-like genes that have been shown to be functionally active. The UL146 gene is highly diverse among sequenced clinical isolates, which suggests that this CXC chemokine is either prone to sequence evolution and/or is genetically responding to environmental pressures. Previously, the genotype GT5 (Tx15 – Heo J. Immuno 2015) has been shown to express a CXCR2 selective chemokine. The current manuscript further characterizes GT5 by determining the N’terminus by proteomics, which revealed that the full-length protein aa1-97 is the most active form, which was different than the previous report from 2015. Structural modeling predicts the extended C’terminus to encode a β-hairpin. The paper describes their results of GT5 in binding, receptor usage, GRK activation, and cellular migration. All of these assays are well done and the results are accurately described in the well written manuscript.*

***Authors reply 3.1***

We are happy to hear that reviewer 3 finds our assays well done, the results accurately described and the manuscript well-written.

We would however like to point out that Heo *et al*:

1. did not investigate vCXCL1_GT5_, but the other non-ELR genotype vCXCL1_GT6_ (=Tx15).
2. did not find any activity of vCXCL1_GT6_ on CXCR2 transfected cells,
3. did not show that vCXCL1_GT6_ was *selective* as they only tested this ligand in functional assays on one receptor (CXCR2, where they found no activity) and in binding on CXCR1 and CXCR2. In contrast, we tested vCXCL1_GT5_ on 19 different chemokine receptors (please see our *authors’ reply 2.5* to reviewer 2).

***Part II - Key experiments required for acceptance:*** *The manuscript functionally determined and evaluated the N’terminus for GT5 identifying the most active form. The requirement and function of the C’terminal region is still unknown. The C’terminal region contains a number of basic residues which could implicate it in protein interactions. Using the assays described in this manuscript, analysis of a vCXCL1 mutant containing deletion of the C’terminal domain to mimic IL-8 or other mutations would have been informative and add to the overall impact of the manuscript. Is this region required for binding and activity or does it play an additional, yet to be defined role?*

***Authors reply 3.2***

We agree with reviewer 3 that analysis of a vCXCL1 mutant containing deletion of the C-terminus using the assays in the manuscript would be informative and add to the impact of the manuscript. As explained in our *authors’ reply 2.3*, we therefore decided to express and purify a tailless version of vCXCL1_GT1_. Interestingly, we found the tailless vCXCL1_GT1_ chemokine non-functional in contrast to the wild-type vCXCL1_GT1._ Furthermore, thermal denaturation using nano differential scanning fluorimetry of the wild-type and tailless vCXCL1_GT1_ revealed that the wild-type chemokine had a melting curve with an apparent Tm of 66.4 ˚C, while the tailless version was unfolded from 20-95 ˚C. Thus, one of the functions of the C-terminus of vCXCL1_GT1_ seems to be to stabilize the chemokine fold of the protein.

*Changes in the manuscript due to this reply follow after all our replies to the comments from the reviewers.*

*The utility of CXCR2 but not CXCR1 by UL146 GT5 is interesting and previously shown by Heo et al. In Figure 9, the authors have aligned GT1 and GT5 with IL8/CXCR2 and suggest that there are contacts R278/R208 and R212 that may allow non-ELR contacts. But what makes CXCR1 so different? Comparative modeling for CXCR1 and CXCR2 may provide insight into this unique feature especially if the structures for GT1 and GT5 are added to this analysis. Similarly, could the amino acid differences between GT1 and GT5 be used as a roadmap to identify the specific differences necessary for the effects of differential binding of CXCR1/2?*

[Again we must draw the attention of the editors and reviewers to the fact that Heo *et al.* did not investigate vCXCL1_GT5_, but vCXCL1_GT6_ (=Tx15), which they found unable to activate G proteins and to recruit β-arrestin in CXCR2 transfected cells – please see *authors reply 2.5]*

***Authors reply 3.3***

Liu et al. in their Nature article from 2020 (ref 53) present cryo-electron microscopy structures of CXCR2 bound to CXCL8 and an antagonist respectively. They also try to answer the question why CXCL6 and CXCL8 bind CXCR1 and CXCR2, while the other human ELR chemokines (CXCL1, 2, 3, 5 and 7) only target CXCR2 by using the structures of CXCR2 to model the CXCR1/CXCL8 interaction. They conclude that the N-terminus (between the PP and PC motifs) of CXCR2 mainly contain hydrophobic residues, while the same region of the N-terminus of CXCR1 contain polar [acidic] residues. They argue that this probably leads CXCR2 to be more promiscuous than CXCR1 with regard to the binding of ELR chemokines and they note that only the N-loops of the CXCL6 and CXCL8 chemokines contain charged residues [in the first part and basic] that can be recognized by CXCR1. Hence, the N-loop of the human ELR chemokines is suggested by the authors’ to be the key to why CXCR1 is different from CXCR2.

Can the suggested dependence of charged residues in the N-loop of human ELR chemokines for CXCR1 binding be transferred to the vCXCL1 chemokines? We think that the reality is more complex. All 14 genotypes except one contain charged residues in their N-loops including vCXCL1_GT1_, vCXCL1_GT4_, vCXCL1_GT5_ and vCXCL1_GT6_ (Fig 1 and Fig S2 in the manuscript), which would suggest CXCR1 targeting; however, our experimental data show that this is only true for vCXCL1_GT1_. The rest of the chemokine fold and the C-terminus could definitely also play a role.

We think that the Rosetta models of the 14 genotypes could be a valuable tool to address this question, but not alone. They need to be combined with crystal/NMR/cryoEM structures of CXCR1 and some of the viral vCXCL1s preferably bound to CXCR1/CXCR2. It is our hope that this manuscript could inspire and be a stepping stone for future work from us and others that could generate the data needed to answer the key question raised by the reviewer.

*The authors pose the question about why there are 14 different known variants of UL146. In Figure 10 and in the discussion the authors provide their explanation about why HCMV encodes at least 14 different genotypes for UL146. The human sequence variation for CXCR1 and 2 is very minimal, thus host variation in CXCR1 and CXCR2 probably does not account for the driving evolutionary changes in UL146. In fact, while basic motifs are shared there is quite a bit of difference between the different genotypes of UL146. In light of this, does the suggest that the protein may be doing something else or that the minor changes are important for alterations in signaling through these receptors? A more in-depth characterization of this is really necessary to try and pick apart whether UL146 is an evolutionarily active hotspot or whether the genetic variation is/was required for viral success in the human population.*

***Authors reply 3.4***

Reviewer 3 brings forward an important question. Actually, we wanted to answer a similar question in the present manuscript: does the non-ELR vCXCL1_GT5_ target other chemokine receptors than CXCR1 and CXCR2 because of its unique N-terminus compared to the other vCXCL1s? Therefore, we decided to test vCXCL1_GT5_ on 19 chemokine receptors. Do we think that the 14 rather different vCXCL1 proteins take advantage of differential signaling through CXCR1 and CXCR2? In our minds, it would be hard to envision 14 different signals arising from the same receptor. We find it more likely that the 14 different chemokines interact with different GAGs or different unknown protein partners. Thus, finding possible interaction partners is key to answer the question posed by reviewer 3.

*Do any of the other UL146 mutants display the same GT5 phenotype with binding CXCR2 and not 1? How about GT6? GT7 through GT14 contain a four amino acid deletion in the N’loop region, which is typically involved in receptor binding, this might also be an interesting group to assess receptor usage.*

***Authors reply 3.5***

Our study is the first that compares activation of different vCXCL1s on both CXCR1 and CXCR2. As a result of the question raised by the reviewer, we decided to expand the manuscript by expressing the other non-ELR chemokine vCXCL1_GT6_ and another ELR chemokine vCXCL1_GT4_ and test them in parallel with vCXCL1_GT1_ and vCXCL1_GT5_ in IP-accumulation assays on cells either expressing CXCR1 or CXCR2. vCXCL1_GT4_, vCXCL1_GT5_ and vCXCL1_GT6_ were found to activate CXCR2, but not CXCR1, while vCXCL1_GT1_ activated both receptors. Regarding binding Heo *et al.* found low affinity binding of vCXCL1_GT1_, vCXCL1_GT2_ and vCXCL1_GT14_ to CXCR1 and no binding of vCXCL1_GT6-13_ to this receptor at all.

As reviewer 3 points out, exploring the significance of N-loop differences for receptor activation could form the basis for interesting future projects. Even more so as Liu et al. (ref 53) suggest that the N-loop of human ELR chemokines is key to understanding the selectivity towards CXCR1 or/and CXCR2 (*please see our authors reply 3.3*). As discussed in our manuscript (Fig 1, Fig 2 and Table S1), it is not only the N-termini, glycosylation patterns and C-termini that show substantial differences, but also the N-loops (due to shorter N-loops in certain genotypes, as pointed out by the reviewer) and the considerable differences seen in charged residues (Fig 1). In our eyes, other important projects would be to determine whether there are differences in GAG and protein interaction partners between the 14 genotypic proteins.

*Changes in the manuscript due to this reply follow after all our replies to the comments from the reviewers.*

**Part III – Minor Issues: Editorial and Data Presentation Modifications:** See above

**Changes to the manuscript due to the added data and the reviewers´comments:**

I. **Additions due to author reply 2.2 (*NMR structure of the vCXCL1_GT1_ tail*):**

*Results:*

*Figure 3 addition of panel G, H and I*

And the following paragraph

“*To assess the reliability of the molecular modeling, we used Nuclear Magnetic Resonance (NMR) to assign the backbone chemical shifts of vCXCL1_GT1_. A heteronuclear single-quantum coherence (HSQC) spectrum showed well-dispersed peaks indicative of a folded protein, including the shifts corresponding to the extended C-terminus (Figure 3G). Using TALOS+, we parsed secondary structure elements in the extended vCXCL1_GT1_ C-terminus based on the 1H, 13Cα, 13Cβ, 13C′, and 15N chemical shifts [31]. The TALOS+ analysis predicted β-strands for residues 72-79 and 85-94 with high confidence, matching the consensus location of the β-hairpin in the Rosetta models (Fig 3H). Additionally, high {1H}-15N heteronuclear NOE values (>0.6) in the C-terminus suggested that the vCXCL1_GT1_ protein backbone in this region is well ordered (Figure 3I).* “

*Discussion:*

*“…..and the identification of two stable β-strands by secondary NMR chemical shift analysis of five atoms (HN, Cα, Cβ, CO, N) for the last 45 residues of vCXCL1_GT1_ in TALOS analysis support the existence of this unique secondary structure.“*

*Materials and methods:*

“***Nuclear magnetic resonance (NMR)***

*NMR experiments were performed on a Bruker DRX 600 equipped with a 1H/15N/13C cryoprobe and a SampleJet robot for automated NMR screening of compounds in 96-well sample racks. NMR samples contained 90% H20, 10% D20, and 0.02% NaN3, with 25 mM D13 2-ethanesulfonic acid (MES) at pH 6.8. 1H, 15N, and 13C resonance assignments for vCXCL1 GT1 were obtained at 25°C using the following experiments: 15N-1H HSQC [61] 3D HNCA [62, 63] 3D HNCO [62, 64], 3D HN(CO)CA [62], 3D HNCACB, 3D HN(CA)CO, 3D HCCONH, 3D SE C(CO)NH [65], and 3D HCCH TOCSY [66]. NMR data were processed with NMRPipe [67] and XEASY [68] was used for resonance assignments and analysis of spectra. Backbone φ and ψ dihedral angle constraints and chemical shift guided predictions of secondary structure were generated from shifts of the 1H, 13Cα, 13Cβ, 13C′, and 15N nuclei using the program TALOS+ [31]. Heteronuclear NOE values were measured from an interleaved pair of two-dimensional 15N-1H sensitivity-enhanced correlation spectra recorded with and without a 5-second proton saturation period.”*

II. **Additions due to author replies 2.3, 2.4 and 3.2 (*function of tailless vCXCL1_GT1_ and comparison with human chemokines with long C-termini*)**

*Results:*

*A new figure 8*

*A new Table S1*

and addition of the following paragraph

“***Deletion of the C-terminus of vCXCL1_GT1_ results in solvent exposure of chemokine core tryptophan residues and in a non-functional protein***

*In general, deletion of the C-terminus from human chemokines with long C-termini does not lead to loss of functional activity [21, 22, 35-44] (Table S1). To investigate if this was also the case for vCXCL1_GT1_, we expressed and purified a tailless vCXCL1_GT1_ protein from E. coli where the 24 distal amino acids were deleted, resulting in a 71 amino acid protein (Fig 8A). Interestingly, the tailless vCXCL1_GT1_ did not induce IP3 accumulation through CXCR1 and CXCR2 in contrast to the wild-type vCXCL1_GT1_ (Figure 8B and C). Next, the thermal denaturation of purified wild-type vCXCL1_GT1_ and the tailless vCXCL1_GT1_ was assessed by nano differential scanning fluorimetry (nanoDSF) (Figure 8D and E). Due to the high sensitivity of tryptophan sidechains to their chemical environment, protein unfolding can be detected by monitoring the intensity and ratio of emission at 350 nm to 330 nm [45]. This analysis gave a two-state melting curve with an apparent Tm of 66.4˚C for wild-type vCXCL1_GT1_, whereas tailless vCXCL1_GT1_ was unfolded from 20–95˚C, demonstrating that the C-*terminal β-*hairpin of vCXCL1_GT1_ is required for folding of a stable chemokine core.”*

*Discussion:*

Addition of the following paragraph

“*The thermal unfolding and functional data of the tailless vCXCL1_GT1_ protein suggest that one role of the terminal β-hairpin is to stabilize the chemokine fold (Fig 8D and E), which contrasts NMR structures of tailless versions of human chemokines with extended C-termini, where the chemokine fold is preserved [21, 22, 35, 39, 40, 46] . Furthermore, additional functions are likely. Documented roles for the unstructured extended C-termini of human chemokines include antifungal activity [21] and glycosaminoglycan (GAG) binding [22, 40, 43]. Thus, a high content of basic residues in the C-terminus has been linked to GAG binding. The C-termini of CCL21 and CXCL12γ are highly basic (32% and 58%) and deletion of these C-termini results in loss of GAG binding [22, 40, 43] (Table S1). Likewise, the C-termini of vCXCL1_GT10_ (34%) and vCXCL1_GT12_ (27%) also have a high content of basic residues suggesting GAG binding (Table S1). In contrast, the extended C-termini of both some of the human chemokines (XCL1 and CCL16) and some of the viral chemokines (vCXCL1_GT1_, vCXCL1_GT2_ and vCXCL1_GT4_) are significantly less basic, which suggest binding to unidentified proteins such as cell surface receptors or other chemokines (Table S1).”*

Added a line at the bottom left of figure 11 that says

“*Binding to specific GAG epitopes*”

*Materials and methods:*

Addition of the the following paragraphs

*“Genotype 1 UL146 (1-71)*

*ACAGAACTACGATGTAGATGCTTACACAGGAAGTGGCCGCCCAACAAAATTATTCTGGGTAATTATTGGCTGCATCGTGATCCGCGTGGCCCAGGCTGCGACAAGAACGAACATCTTCTCTACCCGGACGGCCGCAAACCGCCGGGCCCGGGCGTGTGCTTGTCGCCGGACCACTTGTTTAGCAAGTGGCTGGATAAGCATAACGATAATCGT”*

and

*“****Thermal Denaturation***

*50 µM of purified vCXCL1_GT1_ and vCXCL1_GT1(1-71)_ in 50 mM MES, pH 6.8 were analyzed using nanoDSF on the Nanotemper Prometheus NT.48. Protein samples and a buffer control were placed in triplicate into single use standard capillaries and heated from 20–95 ˚C with a* *heating rate of 0.5˚C/min. Samples were excited at 280 nm and the F350:F330 ratio was monitored during the run to determine an apparent melting temperature (Tm) for each sample.”*

III. **Additions due to author replies 2.5 and 3.6 (*expanding testing in IP3 accumulation assay with the other non-ELR chemokine (vCXCL1_GT6_) and the ELR chemokine (vCXCL1_GT4_ ) on CXCR1 and CXCR2***):

*Results:*

*Expansion of figure 5 from two panels to six panels*

The following paragraph has been changed from

“*Having established that vCXCL1_GT5_ selectively targets CXCR2, in contrast to vCXCL1_GT1_ that targets both CXCR1 and CXCR2 [8], we decided to investigate the dose/response relationship of G protein activation of the two chemokine receptors using vCXCL1_GT5_ with vCXCL1_GT1_ and CXCL8 for reference in the same IP accumulation assay (Fig 5). On CXCR1, vCXCL1_GT1_ activated G protein signaling with a lower potency than CXCL8, while no activity was seen for vCXCL1_GT5_ or its two N-terminally truncated variants (Fig 5A). On CXCR2, vCXCL1_GT1_ almost matched the activity of CXCL8 while vCXCL1_GT5_ was slightly less potent (Fig 5B), and vCXCL1_GT5_ _(4–97)_ and vCXCL1_GT5 (6–97)_ did not significantly activate the receptor at concentrations below 1 µM. From this dose/response investigation, it was clear that the N-terminally truncated vCXCL1_GT5_ variants had severely inhibited signaling properties compared to the full-length chemokine (Fig 5B), which suggests that the first three amino acids of the N-terminus, Gly-Phe-Gly (GFG), are crucial to the activity of vCXCL1_GT5_. Pretreatment with vCXCL1_GT1_ or vCXCL1_GT5_ prior to stimulating with 10 nM CXCL8 on CXCR1 did not* *result in any dose-dependent inhibition of the CXCL8-mediated response (data not shown), which was expected from the initial antagonism screening (Fig 4C).“*

to

*“Having established that vCXCL1_GT5_ selectively targets CXCR2, in contrast to vCXCL1_GT1_ that targets both CXCR1 and CXCR2 [9], we decided to investigate the dose/response relationship of G protein activation of the two chemokine receptors using two ELR vCXCL1s (genotype 1 and genotype 4) and the two non-ELR vCXCL1s (genotype 5 and genotype 6) with CXCL8 for reference (Fig 5A-D). Additionally, the two N-terminally truncated vCXCL1_GT5_ variants were tested (Fig 5E and F). On CXCR1, vCXCL1_GT1_ activated G protein signaling with a lower potency than CXCL8, while no activity was seen for vCXCL1_GT4_ (Fig 5A). On CXCR2, vCXCL1_GT1_ and vCXCL1_GT4_ matched the activity of CXCL8 (Fig 5B). The two non-ELR vCXCL1s (vCXCL1_GT5_ and vCXCL1_GT6_) were, like the ELR chemokine vCXCL1_GT4_, unable to activate CXCR1 (Fig 5C), but activated CXCR2 to the same extent although with lower potency than CXCL8 (Fig 5D). The two N-terminally truncated vCXCL1_GT5_ variants showed the same pattern of activation as vCXCL1_GT5_, but did not significantly activate the receptor at concentrations below 1 µM (Fig 5E and 5F). Pretreatment with vCXCL1_GT1_ or vCXCL1_GT5_ prior to stimulating with 10 nM CXCL8 on CXCR1 did not result in any dose-dependent inhibition of the CXCL8-mediated response (data not shown), which was expected from the initial antagonism screening (Fig 4C).*

*From this dose/response investigation, it was clear that the N-terminally truncated vCXCL1_GT5_ variants had severely inhibited signaling properties compared to the wild-type chemokine (Fig 5F), which suggests that the first three amino acids of the N-terminus, Gly-Phe-Gly (GFG), are crucial to the activity of vCXCL1_GT5_. As expected, the two tested ELR vCXCL1s followed the same pattern of receptor activation as human ELR chemokines in either selectively targeting CXCR2 (vCXCL1_GT4_) or targeting both CXCR1 and CXCR2 (vCXCL1_GT1_).“*

*Discussion:*

The following sentences have been added:

*“ The importance of CXCR1 and CXCR2 as receptor targets for this HCMV-encoded family of chemokines is further supported by the fact that both vCXCL1_GT4_ and vCXCL1_GT6_ activated CXCR2 selectively (Fig 5B and 5D).“*

*and*

*“Thus, in contrast to our findings for vCXCL1_GT6_ and the analogous non-ELR chemokine vCXCL1_GT5_, they did not see any activity of vCXCL1_GT6_.”*

*Materials and methods:*

The following paragraphs have been added:

*“****Prokaryotic expression and purification of UL146 – Protocol 2 (Wisconsin)***

*Sequences of vCXCL1 genotypes 1, 4, 5, and 6 were codon-optimized for E. coli expression and ordered from GenScript. Constructs were cloned into a pET28a-6xHis-SUMO3 vector and expressed in BL21 DE3 E.coli. Cells were expressed at 37°C in LB medium and induced with 1 mM isopropyl-β-D-thiogalactopyranoside (IPTG) at an OD600 of 0.6. Cultures continued to grow for 5 and a half hours before bacteria were pelleted by centrifugation and stored at -20°C. For uniform labeling with 15N, cells were grown in M9 media containing 15N-ammonium chloride as the sole nitrogen source. Bacterial pellets were resuspended in ~20 mL of Buffer A (50 mM Na2PO4 (pH 8.0), 300 mM NaCl, 10 mM imidazole, 1 mM phenylmethylsulphonyl fluoride (PMSF), and 0.1% (v/v) 2-mercaptoethanol (BME)) per pellet and lysed via sonication. Lysed cells were clarified at 15,000 x g and the supernatant was discarded. Pellets were resuspended by sonication in ~20 mL of Buffer AD (6 M guanidinium, 50 mM Na2PO4 (pH 8.0), 300 mM NaCl, 10 mM imidazole) and spun down at 18,000 x g for 20 min. Using an AKTA-Start system (GE Healthcare), supernatant was loaded onto a Ni-NTA column equilibrated in Buffer AD. The column was washed with Buffer AD, and proteins were eluted using Buffer BD (6 M guanidinium, 50 mM sodium acetate (pH 4.5), 300 mM NaCl, and 10 mM imidazole). Proteins were refolded overnight via drop-wise dilution into a 12-fold greater volume of Refold Buffer (50 mM Tris (pH 7.6), 50 mM NaCl) with the addition of 20 mM cysteine, and 0.5 mM cystine. Refolded protein was concentrated in an Amicon Stirred Cell concentrator (Millipore Sigma) using a 10 kDa membrane. Concentrated protein was added to 3.5 kDa dialysis tubing with the addition of ULP1 to cleave the N-terminal 6xHis-SUMO3-tag and dialyzed at 25˚C against Refold Buffer overnight. The AKTA-Start system was used to load cleaved proteins onto a SP FastFlow cation exchange column equilibrated in Refold Buffer. The column was washed with Refold Buffer, and proteins were eluted using Elution Buffer (50 mM Tris (pH 7.6), 1 M NaCl). vCXCL1 proteins were separated from reduced or aggregated species by reversed-phase high performance liquid chromatography, lyophilized, and stored at -80°C for further use. Purity and identity of all proteins were confirmed by electrospray ionization mass spectrometry using a Thermo LTQ instrument, and SDS-PAGE with Coomassie staining.*

*Recombinant proteins of vCXCL1_GT1_ and vCXCL1_GT5_ were produced by both protocol 1 and 2, and when compared, they activated G proteins through CXCR1 and CXCR2 to exactly the same extent in the IP3 accumulation assay.*

*The following gene sequences were used for expression in protocol 2:*

*Genotype 1 UL146 (Toledo strain, 1-95)*

*ACAGAACTACGATGTAGATGCTTACACAGGAAGTGGCCGCCCAACAAAATTATTCTGGGTAATTATTGGCTGCATCGTGATCCGCGTGGCCCAGGCTGCGACAAGAACGAACATCTTCTCTACCCGGACGGCCGCAAACCGCCGGGCCCGGGCGTGTGCTTGTCGCCGGACCACTTGTTTAGCAAGTGGCTGGATAAGCATAACGATAATCGTTGGTATAACGTAAATATCACCAAGAGCCCGGGTCCGCGCCGTATTAACATCACCCTGATCGGTGTCCGTGGT*

*Genotype 1 UL146 (1-71)*

*ACAGAACTACGATGTAGATGCTTACACAGGAAGTGGCCGCCCAACAAAATTATTCTGGGTAATTATTGGCTGCATCGTGATCCGCGTGGCCCAGGCTGCGACAAGAACGAACATCTTCTCTACCCGGACGGCCGCAAACCGCCGGGCCCGGGCGTGTGCTTGTCGCCGGACCACTTGTTTAGCAAGTGGCTGGATAAGCATAACGATAATCGT*

*Genotype 4 UL146 (NT strain)*

*ACCGAGCTGCGTTGCAAATGCGCAGGTGGCCAAAGCTGGCATCCGAGAGGCAAGTGGCCAACCAAACATCATTGGTTGGAGTGCTACCCGCCGAGTGGAAACTGTCCGGCGGGCGAATTGCTGATTTATTTCGAGGAACACAACTGGAGCCCGAAGTGCGTTCATGTTCACAATCCGTTTGGCCAAAAATTCATGAGCAAGTGCGATAAACACGAATGGTTCGAGGTTACCTTCAACAGCACTCGTAAGTACCCGATGATCACCCGCAAGGGCTCGACCAAACCGACCTTCAGCTCCGGCAAA*

*Genotype 5 UL146 (Davis strain)*

*GGTTTTGGCACTGAGGGCAACGGCCGTTGCACGTGCATTGGCTACCACCGCCTGTTAGGTCAACTGCCGCGGGGTACTTTCTGGCTCGGCCATCTGCCGCCAGGTAGCCATTGCCCGAAAGGTCAAGTTATGATCAAGATCGGTCAAGGTCCGATTGTGTGCTTGTCCGACTACCACCCGTTGTCTAAGTGGATGTACGGCAACCACAAAAGCGGCAGCGAAACCTGGCTGCAAATCAAAATGGAAGGTCCTAGAAATGCTACCGTGGTGCAGCGTAGCAATACGCGTCCG*

*Genotype 6 UL146 (ML1 strain)*

*GGTCTGGGTAGCGAGGGGAACGGTCGTTGTACCTGTGTCGGTTATCACCGTTTTGATAAGCAGCTGCCACGCGGTACGATCTGGCTGGGTCACCGCCCTCCGGGCCCACACTGTCCGCGCGGTGACGTTCTGATGAAACTGGGTGAACAGCCGACGGTGTGTTTGAGCGACCACCACCCACTGTCCAAATGGATGTATCGTCATCACGGCTCCGACACCGAAATCTGGTTCCAGATTGAGTTTAAAGGTCCGCAGAATACCAAGGTGGTTTCTAAGTCCTTTACCCCGCCGTCA“*

IV. **Other changes:**
i) We have removed “predicted” from the title as we have now added in vitro data that support the existence of the β-hairpin. The purpose was to shorten the title; however, if the editors prefer the original title, we have no objections.
ii) We have adapted the abstract to include the added data.
iii) We have adapted the last paragraph of the introduction, the first and last paragraph of the discussion in order to reflect the added data.
iv) We have made smaller changes to improve the language in some places.
v) The number of references has been increased in the M&Ms, results and discussion sections as a result of the additional experiments.
vi) We have added a new supplemental figure S2 Fig. and removed data not shown
vii) We have removed the financial disclosure statement from the manuscript as this information has to be pasted in a box in the submission system.
