# Supplementary material for: The non-ELR CXC chemokine encoded by human cytomegalovirus UL146 genotype 5 contains a C-terminal β-hairpin and induces neutrophil migration as a selective CXCR2 agonist
Source: PLoS Pathog. 2022 Mar 10;18(3):e1010355. doi: 10.1371/journal.ppat.1010355 (PMC8939814; doi:10.1371/journal.ppat.1010355)
Supplement: Attachment — Submitted filename: 220207 Answer to reviewers second round.docx [file ppat.1010355.s006.docx]

**Answers to the reviewers:**

***Reviewer 1:***

***Part I - Summary:*** *The manuscript by Berg et al describes comprehensive analysis of the UL146 isotype vCXCL1-GTA5, which lacks the classic ELR motif in the N-terminus. The authors convincingly show that vCXCL1-GTA is a selective agonist for CXCR2 only, not CXCR1 as other isotypes of vCXCL1, such as GT1, using multiple readouts that include IP accumulation, beta-arrestin recruitment, and neutrophil migration. The authors were responsive to the previous reviewer comments and the overall article is suitable for publication.*

***Authors reply 1.1:***

We are happy to hear that Reviewer 1 finds we have been responsive to the reviewers’ comments.

**Part II - Key experiments required for acceptance**: No response

**Part III – Minor Issues: Editorial and Data Presentation Modifications:**

1. *Key words – consider ELR motif instead of ELR*

***Authors reply 1.2:***

We have changed “ELR” to “ELR motif”.

2. *Pg 49, lines 737-742 It’s a lot of work to clone all and express all the chemokine receptors. Was this reported elsewhere that can be cited here? Also, was there a myc/His or similar tag on the receptor to verify expression? The ligand responses are convincing, but it seems like at least commenting on how receptor expression was verified would be helpful.*

***Authors reply 1.3:***

For the functional assays, we chose to use untagged receptors in order to eliminate any potential effect on ligand binding of the tags. Receptor expression was verified when cells could produce a signal (dose response) upon stimulation by an agonist of the expressed chemokine receptor (and this signal was not present in the cell line without receptor expression). Our lab houses a large library of tagged and untagged chemokine receptor constructs in expression plasmids. We have previously tested both FLAG-tagged and untagged versions of the receptors in parallel with no significant difference in activity, where the FLAG-tagged receptor expression was confirmed by ELISA. We have inserted two references as suggested by the reviewer and expanded the information on the receptor constructs used for increased transparency.

Page 49 line 737-742 in the tracked changes version corresponds to page 40 line 629-634 in the clean version.

As a result, we have changed the following paragraph from:

“*For all chemokine receptors used, the human wild type receptor cDNA was cloned from a spleen-derived cDNA library into the expression vector pcDNA3.1(+) (Invitrogen). The chimeric G protein GαΔ6qi4myr (Gqi4myr) was kindly provided by Evi Kostenis (University of Bonn). The Rluc8-arrestin3-Sp1 and mem-citrine-SH3, GRK2, and GRK6 plasmids were kindly provided by Jonathan Javitch (Columbia University). All constructs were verified by restriction endonuclease digestion and DNA sequencing*.”

to:

“*For all chemokine receptors used, the human wild type receptor cDNAs without tags were cloned into the expression vector pcDNA3.1(+) (Invitrogen). The following restriction sites were used for insertion: CXCR1, CXCR2, CXCR3, CXCR4, CXCR6, XCR1, CX3CR1, CCR2, CCR6 and CCR7 (HindIII-XhoI); CXCR5 and CCR5 (HindIII-BamHI); CCR1, CCR3, CCR4, CCR9 and CCR10 (EcoRI-XhoI); CCR8 (HindIII-EcoRI); US28 (EcoRI-NotI). All constructs were verified by restriction endonuclease digestion and DNA sequencing. The chimeric G protein GαΔ6qi4myr (Gqi4myr) inserted into pcDNA3.1(+) was kindly provided by Evi Kostenis (University of Bonn) [60]. The Rluc8-arrestin3-Sp1 and mem-citrine-SH3, GRK2, and GRK6 plasmids were kindly provided by Jonathan Javitch (Columbia University) [61]. Receptor expression was verified by a functional response, i.e. when transfected cells responded upon stimulation by a natural chemokine agonist of the expressed chemokine receptor (for details see figure 4) while non-transfected cells showed no response to the same chemokine. ”*

*3.* *Pg. 61, lines 1023 and 1026 -cell number is listed as 150.000 – us a comma or scientific notation (150,000 or 1.5 x 10^5), this is confusing as written*

***Authors reply 1.4:***

We agree and have corrected the sentence with a comma.

***Reviewer 2:***

***Part I - Summary****: In this revised manuscript, the authors have been responsive to the many of the previous reviews by adding several new experiments, including vCXCL1tailless, a C terminal truncation, and an additional NRG motif (vCXCL1GT6). These important controls make this a much more robust study. In this process they have show another NRG vCXCL1 can stimulate CXCR2 and that with out the COOH tail, the protein miss folds. That is a very interesting finding.*

***Authors reply 2.1:***

We are happy to hear that Reviewer 2 finds that we have been responsive to many of the previous reviews by adding results from several new experiments leading to a more robust study. Furthermore, we are thrilled that Reviewer 2 finds it interesting that another vCXCL1 (vCXCL1_GT6_) can stimulate CXCR2 and that vCXCL1_GT1_ without the C-tail misfolds.

**Part II - Key experiments required for acceptance**: *Below are some important experiments that would make this manuscript more complete:*

1) *The addition of MALDI data on the three new proteins in this submission vCXCL1GT4, vCXCL1GT6, and vCXCL1GT1Tailless to show the proper size and (potential) glycosylations (i.e., vCXCL1GT4 is O linked, Tailless vCXCL1GT1, vCXCL1GT5 is N linked, vCXCL1GT6 is non-glycosylated). This would expand out the Fig. 2.*
2) *For completion /understanding whether there are differences in b arrestin signaling (Fig. 6) and migration (Fig. 7). They show b arrestin and activation with GT1, 5, but needs GT4, 6 in Fig. 6 in Fig. 7. Who knows how well these other viral chemokines affect this signaling. Could it be biased agonism?*

***Authors reply 2.2:***

The findings in the current manuscript open the door to many questions that need to be addressed in future studies, to understand why CMV encodes this unique group of diverse chemokines with similar receptor targets. One of these questions is whether the predicted glycosylation patterns in figure 2 (a panel inserted as requested by Reviewer 3) can be confirmed by experiments as put forward by Reviewer 2. We have made experiments that support the predicted glycosylation patterns for vCXCL1_GT1_ and vCXCL1_GT5_. We agree with Reviewer 2 that it would be interesting to make similar experiments for vCXCL1_GT4_ and CXCL1_GT6_.

Another question raised by Reviewer 2 is whether vCXCL1_GT4_ and vCXCL1_GT6_ also showed beta-arrestin activation and could induce chemotaxis of neutrophils and cells transfected with CXCR1 and CXCR2 as we have shown for vCXCL1_GT1_ and vCXCL1_GT5_.

To summarize the added data after the first review round:

In the first revision, we included experiments requested by the reviewers that

i) confirmed the existence of the beta-hairpin of C-terminus by NMR, which we found by *in silico* modelling.
ii) described that the C-terminal truncated vCXCL1_GT1_ was non-functional and unfolded.
iii) showed that the other non-ELR chemokine vCXCL1_GT6_ also was able to activate CXCR2 like we have shown for vCXCL1_GT5_.

We agree with Reviewer 2 that these additional experiments have resulted in a strengthened version of the original manuscript. However, the time taken to generate and incorporate the data for this expansion of the manuscript took 6 months. Another round of experiments as suggested by Reviewer 2 could easily take 6-12 months (to generate the constructs for these experiments, to make the expression in eukaryotic cells, to purify the proteins and then do the MALDI-TOF- and deglycosylation experiments and, in parallel, make the beta-arrestin activation- and chemotaxis assays). Considering the required time investment, we do not believe that this is a reasonable request as the extra experiments *would not change or affect* the main findings of our current work. Furthermore, we also have an obligation to share the findings of the current manuscript (which Reviewer 2 and we find interesting) with the scientific community without unnecessary delay. We think that the best way to bring science forward is to share new findings and, in that way, provide others with the opportunity to pose questions based on these findings and answer them.

*3)	There needs to be an analysis in the discussion about their findings with tailless vCXCL1GT1 since this is a novel finding. Are there any other chemokine examples where and truncations in the C terminus affected function and/or folding?*

***Authors reply 2.3:***

This question has already been addressed in Table S1, where we have gathered the information available in the literature on the effects of C-terminal truncation on preservation of the chemokine fold and functional activity of the seven human chemokines with long C-termini. Please see the columns to the right headed “*preservation of chemokine fold*” and “*retains functional activity*”.

We made our best effort to describe the key points in Table S1 in a paragraph in the discussion. However, giving this paragraph a second look, we agree with Reviewer 2 that we can expand it further and introduce a few additional references from Table S1.

Thus, the existing paragraph (with requested information in yellow) has been changed from:

*“While an extended C-terminal tail is also found in some human chemokines, these lack secondary structure elements in contrast to these HCMV-encoded chemokines [19-22] (S1 Table). The presence of the β-hairpin in a majority of the 14 genotypes implies conserved but unknown functions. The thermal unfolding and functional data of the tailless vCXCL1_GT1_ protein suggest that one role of the terminal β-hairpin is to stabilize the chemokine fold (Fig 8D and E), which contrasts NMR structures of tailless versions of human chemokines with extended C-termini, where the chemokine fold is preserved [21, 22, 35, 39, 40, 46]. Furthermore, additional functions are likely. Documented roles for the unstructured extended C-termini of human chemokines include antifungal activity [21] and glycosaminoglycan (GAG) binding [22, 40, 43]. Thus, a high content of basic residues in the C-terminus has been linked to GAG binding. The C-termini of CCL21 and CXCL12γ are highly basic (32% and 58%) and deletion of these C-termini results in loss of GAG binding [22, 40, 43] (S1 Table). Likewise, the C-termini of vCXCL1_GT10_ (34%) and vCXCL1_GT12_ (27%) also have a high content of basic residues suggesting GAG binding (S1 Table). In contrast, the extended C-termini of both some of the human chemokines (XCL1 and CCL16) and some of the viral chemokines (vCXCL1_GT1_, vCXCL1_GT2_ and vCXCL1_GT4_) are significantly less basic, which suggest binding to unidentified proteins such as cell surface receptors or other chemokines (S1 Table).”*

to (with the additions in bold):

“*While an extended C-terminal tail is also found in some human chemokines, these lack secondary structure elements in contrast to these HCMV-encoded chemokines [19-22] (S1 Table). The presence of the β-hairpin in a majority of the 14 genotypes implies conserved but unknown functions. The thermal unfolding and functional data of the tailless vCXCL1_GT1_ protein suggest that one role of the terminal β -hairpin is to stabilize the chemokine fold (Fig 8D and E), which contrasts NMR structures of tailless versions of human chemokines with extended C-termini* ***(XCL1,*** ***CCL21, CCL25, CCL28 and CXCL12γ),*** *where the chemokine fold is preserved [21, 22, 35, 39, 40, 46 and* ***S1 Table]*** ***and which retain functional activity [21, 22, 35, 36, 39, 40, 41, 42, 43, 44 and S1 Table].*** *Furthermore,* ***in addition to stabilization of the chemokine fold*** *other functions* ***of the β-hairpin*** *are likely. Documented roles for the unstructured extended C-termini of human chemokines include antifungal activity [21] and glycosaminoglycan (GAG) binding [22, 40, 43]. Thus, a high content of basic residues in the C-terminus has been linked to GAG binding. The C-termini of CCL21 and CXCL12γ are highly basic (32% and 58%) and deletion of these C-termini results in loss of GAG binding [22, 40, 43] (S1 Table). Likewise, the C-termini of vCXCL1_GT10_ (34%) and vCXCL1_GT12_ (27%) also have a high content of basic residues suggesting GAG binding (S1 Table). In contrast, the extended C-termini of both some of the human chemokines (XCL1 and CCL16) and some of the viral chemokines (vCXCL1_GT1_, vCXCL1_GT2_ and vCXCL1_GT4_) are significantly less basic, which suggest binding to unidentified proteins such as cell surface receptors or other chemokines (S1 Table).*”

**Part III – Minor Issues: Editorial and Data Presentation Modifications:**

*These statements are not true:*

1) *a. Line 101 “A non-ELR CXC chemokine activates CXCR2 and adopts a unique β-hairpin have been observed for several UL146 genotypes [10], but, apart from genotype 1, the 13 104 remaining genotypes are yet to be characterized in detail.”*

*This is exactly what reference 10 did in that manuscript.*

***Authors reply 2.4:***

We agree with Reviewer 2 that the last part of the sentence is not specific enough, so we have specified that part.

Thus, the following paragraph in the introduction have been changed from:

“*Differences of variable importance in CXCR1/CXCR2 binding affinity, signaling potency, and chemotactic properties have been observed for several UL146 genotypes [10], but, apart from genotype 1, the 13 remaining genotypes are yet to be characterized in detail*.”

to:

“*Differences of variable importance in CXCR1/CXCR2 binding affinity, CXCR2 signaling potency, and chemotactic properties have been observed for several UL146 genotypes [10], but, apart from genotype 1 [9], the 13 remaining genotypes have yet to be characterized for functional activity against the remaining 17 chemokine receptors including CXCR1*.”

2) *b. Line 324 “Previous studies have demonstrated an effect of vCXCL1GT1 on G protein signaling, but as other pathways have not been investigated”. Again reference 10 looked at signaling of the different viral chemokines including b arrestin signaling.*

***Authors reply 2.5:***

We agree with reviewer 2 that this sentence is not entirely correct and apologize for that.

As a result, we have changed the sentence from:

*“Previous studies have demonstrated an effect of vCXCL1_GT1_ on G protein signaling, but as other pathways have not been investigated, we performed a BRET-based β-arrestin recruitment assay for vCXCL1_GT1_ and vCXCL1_GT5_ on CXCR1 and CXCR2 using CXCL8 as a positive control (Fig 6).”*

to:

*“A previous study has demonstrated the ability of several vCXCL1s to recruit β-arrestin through CXCR2 [10]. But in order to investigate the ability of vCXCL1_GT1_ and vCXCL1_GT5_ to recruit β-arrestin by the two receptors, which are targeted by ELR chemokine ligands, we performed a BRET-based β-arrestin recruitment assay for these viral chemokines on CXCR1 and CXCR2 using CXCL8 as a positive control (Fig 6).”*

3) *c. In the discussion: Line 603: “Secondly, by searching for associations between specific viral genotypes and CMV disease, which has the potential to show the effect of the gene variants. Lastly, by performing genome-wide association studies (GWAS) of human hosts infected with HCMV to identify host and viral gene variants associated with disease development.” This statements (and Fig. 11) need to be modified to reflect the literature. UL146 variation has been addressed in both congenital infections (Bale and Demmler publications) and transplantation (Hassan Walker publication) settings so this statement should be modified. They were not able to connect any specific UL146 sequences with specific outcomes. Also, there is very little variability in human CXCR1 and CXCR2 (as mentioned by the previous reviewer) so it is not correct that viral gene variants would develop to infect different “populations” of humans more efficiently. These statements should be modified.*

***Authors reply 2.6:***

We are a little bit confused as to what Reviewer 2 wants to change. We agree with Reviewer 2 that there has been numerous reports of the frequencies of genotypes of polymorphic CMV genes in patient groups with specific CMV diseases, with the majority reporting on children with congenital CMV disease. We find that two important conclusions can be drawn from review of the literature.

1. Reports are limited by small sample sizes and lack of control groups.
2. And, importantly, no links between certain genotypes of polymorphic CMV genes and CMV disease have so far been established.

As to the publications mentioned by Reviewer 2.

- Reviewer 2 refers to the Bale and Demmler publications regarding UL146 and congenital infection. The only publication we can find in PubMed including these two authors on UL146 is a publication in *Journal of Medical Virology from 2008 378: 86-96 by Heo et al. from* *Tim Sparers group*. Here genotypes from the UL144 and UL146 genes from 51 viral isolates from congenitally infected children and 13 viral isolates from children in daycare were investigated. The authors conclude “Even though our study includes a relatively large sample size, we are unable to reach statistically significant conclusions about specific UL146 clades and symptomatic outcomes”.
- The Hassan-Walker publication (*2004 Journal of Medical Virology 74: 573-79*) investigated UL146 genotypes from viral isolates from 5 bone-marrow transplant recipients, 3 renal transplant recipients and 7 seven infants suspected of congenital CMV infection. The authors conclude in the discussion “*No clear evidence exists to associate particular HCMV strains with disease*” and further “*The small number of patients described in this report precludes an analysis of HCMV polymorphisms and clinical* *outcome*”.

Own investigations

- The curiosity regarding the existence of two non-ELR versus twelve ELR CXC-chemokine genes in circulating CMV strains resulted in the data presented in the current manuscript. The same curiosity gave rise to another project, where we wanted to see whether CMV strains encoding the non-ELR genotypes (GT5 and GT6) were associated with congenital CMV disease. We extracted viral DNA from Guthrie cards (stored in the Danish National Biobank since 1982) from 116 newborns diagnosed with congenital CMV disease and urine from 83 children with suspected bacterial infections (controls). We were unable to find an association between any particular UL146 genotype and congenital CMV disease. (Ref: *Berg et al. BMC Infect Dis. 2021; 21(1): 386*).
- In June 2019, we did an in-house analysis of UL146 sequences deposited in GenBank. We found 675 sequences and combined with our 199 sequences mentioned above we were able to identify 365 sequences from children with congenital infection, 28 sequences from hematopoitic stem cell transplant recipients, 24 sequences from kidney transplant recipients and 5 from lung transplant recipients. Furthermore 130 sequences were from immunocompetent persons, which can be regarded as representative for the isolates circulating in the general population and function as controls (of these were 83 from our own study mentioned above).

It is obvious that the number of patients with CMV disease where UL146 have been genotyped are extremely low for the different groups of transplant recipients as well as for controls (47 persons if we exclude our own study).

In conclusion, we agree with Reviewer 2 that no associations have been established between specific CMV genotypes and any particular type of CMV disease.

However, due to the often very small sample sizes and the lack of control groups in the published reports resulting in the very small numbers seen in GenBank, we think it is a line of research that need to be pursued in future projects, which should address these limitations. In response to the point raised by Reviewer 2, we have therefore added a sentence (in yellow) to make this clear and changed the text from:

*“Secondly, by searching for associations between specific viral genotypes and CMV disease, which has the potential to show the effect of the gene variants. Lastly, by performing genome-wide association studies (GWAS) of human hosts infected with HCMV to identify host and viral gene variants associated with disease development.”*

to:

*“Secondly, by searching for associations between specific viral genotypes and CMV disease, which has the potential to show the effect of the gene variants if the lack of large sample sizes and controls groups are addressed. Lastly, by performing genome-wide association studies (GWAS) of human hosts infected with HCMV to identify host and viral gene variants associated with disease development.”*

With this clarification, we do not see the necessity for a change in the bottom right of figure 11. However, if the editor/reviewer finds it necessary we could change

- “*Association studies: CMV disease and UL146 genotypic variants”*

to

- “*Association studies properly powered and with control groups: CMV disease and UL146 genotypic variants*”

4) *Also, there is very little variability in human CXCR1 and CXCR2 (as mentioned by the previous reviewer) so it is not correct that viral gene variants would develop to infect different “populations” of humans more efficiently. These statements should be modified.*

***Authors reply 2.7:***

In our response (authors’ reply 3.4) to Reviewer 3 in the previous review round, we stated that

“Do we think that the 14 rather different vCXCL1 proteins take advantage of differential signaling through CXCR1 and CXCR2? In our minds, it would be hard to envision 14 different signals arising from the same receptor. We find it more likely that the 14 different chemokines interact with different GAGs or different unknown protein partners. Thus, finding possible interaction partners is key to answer the question posed by reviewer 3.”

Furthermore, we do not mention CXCR1 and CXCR2 in the paragraph Reviewer 2 is referring to, which reads

“*Another possible explanation is that encoding several forms of the same immune modulator provides HCMV with the tools to successfully infect and establish infection in a broader variety of hosts, overcoming immunological bottlenecks (Fig 11).”*

On the contrary, we are fully aware of the importance to approach this question with an open mind, as we have tried to do throughout in the discussion and the concluding remarks. But most importantly, we state this explicitly in Figure 11 (bottom left), where we give examples of the possible immunological bottlenecks such as:

*“Bottlenecks - examples*

- *Specific GAG epitopes?*
- *Chemokine receptor usage?*
- *NK-cell receptor variants?*
- *Chemokine protease variants?*
- *Others? “*

5) *d. Line 331: “For CXCR2, both vCXCL1GT5 and vCXCL1GT5 stimulated β-arrestin recruitment at comparable efficacies to CXCL8 but with ~10-fold lower potencies (Fig 6D–F)” Should read both “vCXCL1GT1 and vCXCL1GT5“*

***Authors reply 2.8:***

We thank Reviewer 2 for addressing this typo.
